# *catena*-Poly[[aqua­bis­(4-formyl­benzoato-κ^2^ *O* ^1^,*O* ^1′^)cadmium]-μ-pyrazine-κ^2^ *N*:*N*′]

**DOI:** 10.1107/S1600536813035010

**Published:** 2014-01-11

**Authors:** Fatih Çelik, Nefise Dilek, Nagihan Çaylak Delibaş, Hacali Necefoğlu, Tuncer Hökelek

**Affiliations:** a Department of Chemistry, Kafkas University, 36100 Kars, Turkey; b Aksaray University, Department of Physics, 68100, Aksaray, Turkey; c Department of Physics, Sakarya University, 54187 Esentepe, Sakarya, Turkey; d Department of Physics, Hacettepe University, 06800 Beytepe, Ankara, Turkey

## Abstract

The polymeric title compound, [Cd(C_8_H_5_O_3_)_2_(C_4_H_4_N_2_)(H_2_O)]_*n*_, contains two 4-formyl­benzoate (FB) anions, one pyrazine mol­ecule and one coordinating water mol­ecule; the FB anions act as bidentate ligands. The O atom, the aldehyde H atom and the benzene ring of one of the FB anions are disordered over two positions. The O atoms were freely refined [refined occupancy ratio 0.79 (2):0.21 (2)], while the aldehyde H atoms and the benzene ring atoms were refined with fixed occupancy ratios of 0.8:0.2 and 0.5:0.5, respectively. In the ordered FB anion, the carboxyl­ate group is twisted away from the attached benzene ring (*A*) by 22.7 (8)°. In the disordered FB anion, the corresponding angles are 15.6 (10) and 11.4 (11)° for rings *B* and *B*′, respectively. Benzene rings *A* and *B* are oriented at a dihedral angle of 24.2 (7), *A* and *B*′ at 43.0 (8)°. The pyrazine ring makes dihedral angles of 67.5 (4), 89.6 (7) and 86.2 (7)°, respectively, with benzene rings *A*, *B* and *B*′. The pyrazine ligands bridge the Cd^II^ cations, forming polymeric chains running along the *b*-axis direction. In the crystal, O—H_water_ ⋯ O_carboxyl­ate_ hydrogen bonds link adjacent chains into layers parallel to the *bc* plane. These layers are linked *via* C—H_pyrazine_ ⋯ O_form­yl_ hydrogen bonds, forming a three-dimensional network. π–π interactions [centroid–centroid distances = 3.870 (11)–3.951 (5) Å] further stabilize the crystal structure. There is also a weak C—H⋯π inter­action present.

## Related literature   

For structural functions and coordination relationships of the aryl­carboxyl­ate ion in transition metal complexes of benzoic acid derivatives, see: Nadzhafov *et al.* (1981 ▶); Shnulin *et al.* (1981 ▶). For applications of transition metal complexes with biochemical mol­ecules in biological systems, see: Antolini *et al.* (1982 ▶). Some benzoic acid derivatives such as 4-amino­benzoic acid have been extensively reported in coordination chemistry, as bifunctional organic ligands, due to the varieties of their coordination modes, see: Chen & Chen (2002 ▶); Amiraslanov *et al.* (1979 ▶); Hauptmann *et al.* (2000 ▶). For related structures, see: Hökelek *et al.* (2009 ▶); Sertçelik *et al.* (2013 ▶). For bond-length data, see: Allen *et al.* (1987 ▶).
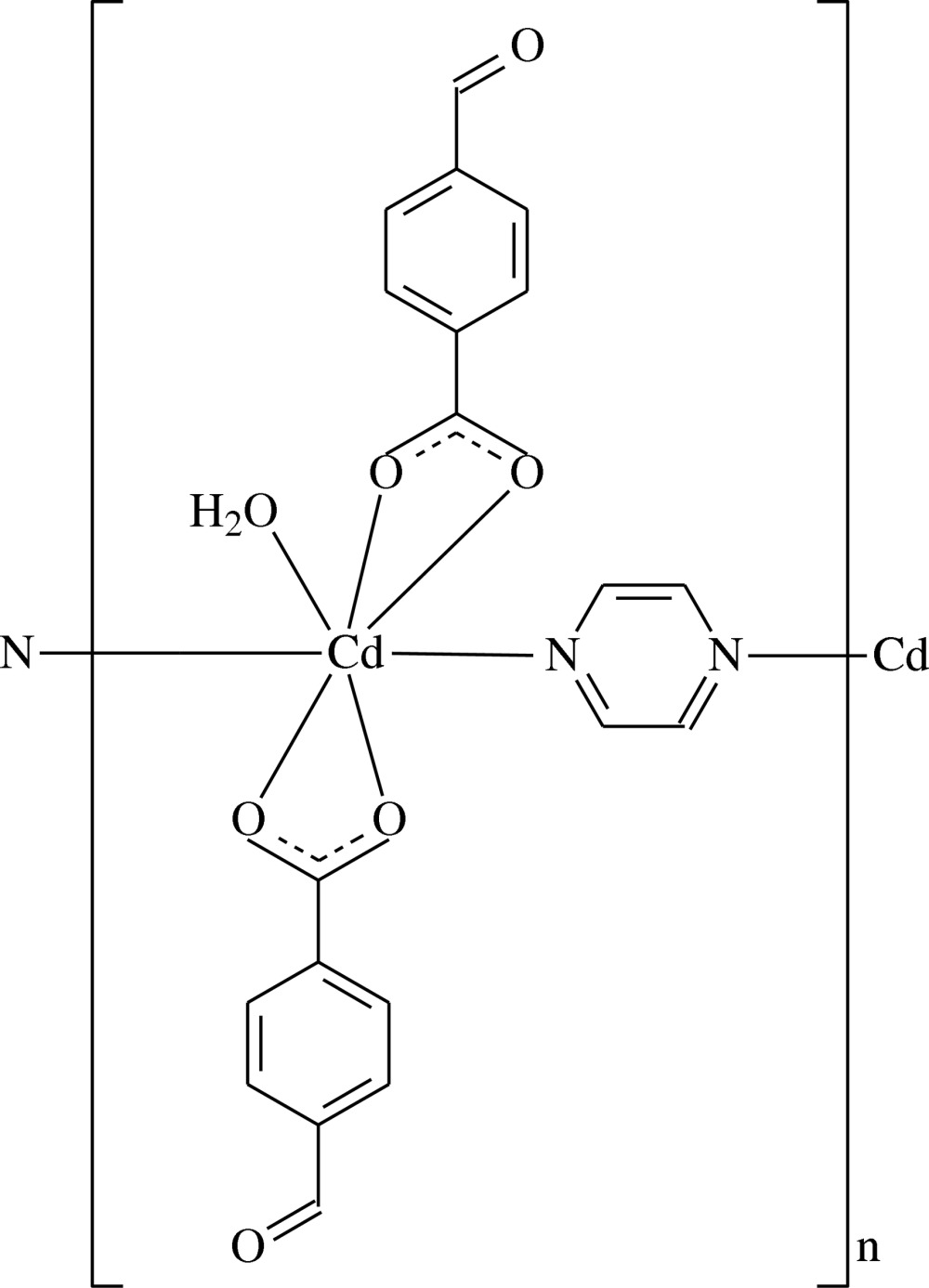


## Experimental   

### 

#### Crystal data  

[Cd(C_8_H_5_O_3_)_2_(C_4_H_4_N_2_)(H_2_O)]
*M* *_r_* = 508.76
Monoclinic, 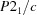

*a* = 22.6016 (5) Å
*b* = 7.4947 (2) Å
*c* = 11.9196 (3) Å
β = 99.673 (4)°
*V* = 1990.38 (9) Å^3^
*Z* = 4
Mo *K*α radiation
μ = 1.14 mm^−1^
*T* = 294 K
0.45 × 0.35 × 0.15 mm

#### Data collection  

Bruker SMART BREEZE CCD diffractometer
Absorption correction: multi-scan (*SADABS*; Bruker, 2012 ▶) *T*_min_ = 0.625, *T*_max_ = 0.842
40178 measured reflections
3587 independent reflections
3497 reflections with *I* > 2σ(*I*)
*R*_int_ = 0.048

#### Refinement  

*R*[*F* ^2^ > 2σ(*F* ^2^)] = 0.059
*wR*(*F* ^2^) = 0.144
*S* = 1.35
3587 reflections
287 parameters
3 restraints
H atoms treated by a mixture of independent and constrained refinement
Δρ_max_ = 1.77 e Å^−3^
Δρ_min_ = −1.85 e Å^−3^

### 

Data collection: *APEX2* (Bruker, 2012 ▶); cell refinement: *SAINT* (Bruker, 2012 ▶); data reduction: *SAINT*; program(s) used to solve structure: *SHELXS97* (Sheldrick, 2008 ▶); program(s) used to refine structure: *SHELXL97* (Sheldrick, 2008 ▶); molecular graphics: *ORTEP-3* for Windows (Farrugia, 2012 ▶); software used to prepare material for publication: *WinGX* publication routines (Farrugia, 2012 ▶) and *PLATON* (Spek, 2009 ▶).

## Supplementary Material

Crystal structure: contains datablock(s) I, global. DOI: 10.1107/S1600536813035010/su2679sup1.cif

Structure factors: contains datablock(s) I. DOI: 10.1107/S1600536813035010/su2679Isup2.hkl

CCDC reference:

Additional supporting information:  crystallographic information; 3D view; checkCIF report

## Figures and Tables

**Table 1:** Hydrogen-bond geometry (Å, °) *Cg*1 is the centroid of the pyrazine ring N1/N2/C17—C20.

*D*—H⋯*A*	*D*—H	H⋯*A*	*D*⋯*A*	*D*—H⋯*A*
O7—H72⋯O5^i^	0.82 (2)	2.10 (6)	2.727 (7)	133 (7)
C18—H18⋯O6*A* ^ii^	0.93	2.52	3.394 (14)	157
C19—H19⋯O3^iii^	0.93	2.43	3.085 (10)	127
C8—H8⋯*Cg*1^iv^	0.93	2.93	3.691 (10)	147

Symmetry codes: (i) 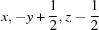; (ii) 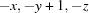; (iii) 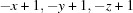; (iv) 
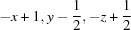
.

**Fig. 1.**
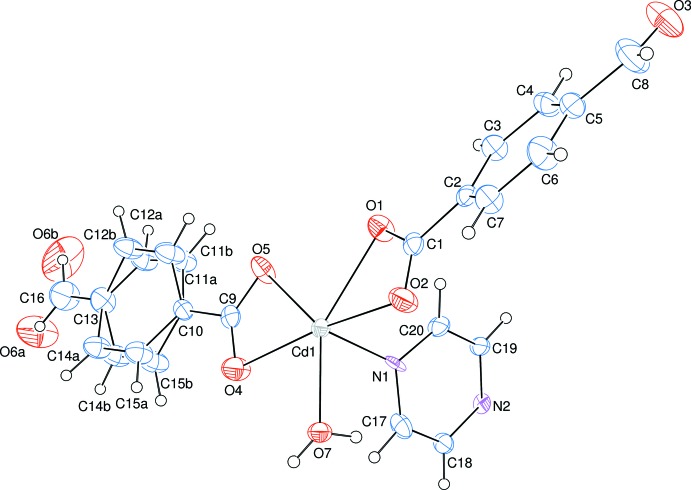
The asymmetric unit of the title molecule, with atom labelling. Displacement ellipsoids are drawn at the 50% probability level.

**Fig. 2.**
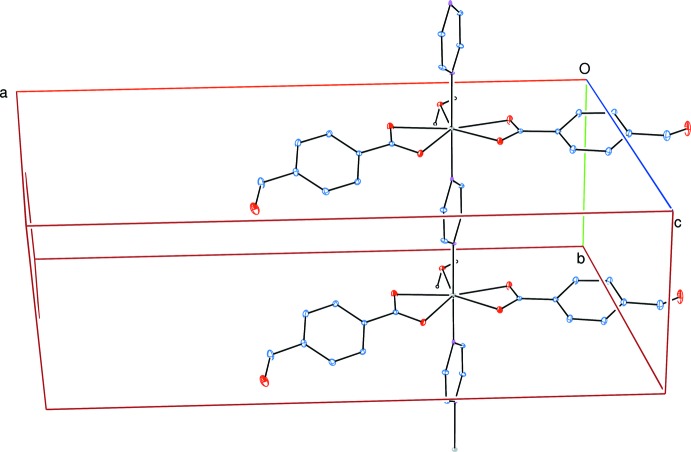
Part of the polymeric chain of the title compound. Only the water H atoms and the major components of the disordered aldehyde and benzene ring are shown.

## supplementary crystallographic information

### 1. Comment

The structural functions and coordination relationships of the arylcarboxylate ion in transition metal complexes of benzoic acid derivatives change depending on the nature and position of the substituent groups on the benzene ring, the nature of the additional ligand molecule or solvent, and the medium of the synthesis (Nadzhafov *et al.*, 1981; Shnulin *et al.*, 1981). Transition metal complexes with biochemically active ligands frequently show interesting physical and/or chemical properties, as a result they may find applications in biological systems (Antolini *et al.*, 1982). Some benzoic acid derivatives, such as 4-aminobenzoic acid, have been extensively reported in coordination chemistry, as bifunctional organic ligands, due to the varieties of their coordination modes (Chen & Chen, 2002; Amiraslanov *et al.*, 1979; Hauptmann *et al.*, 2000). The title compound was synthesized and its crystal structure is reported on herein.

The asymmetric unit of the title polymeric compound contains one Cd^II^ ion, two 4-formylbenzoate (FB) anions, one pyrazine molecule and one coordinated water molecule; the FB anions act as bidentate ligands (Fig. 1). The pyrazine ligands bridge the adjacent Cd^II^ ions forming polymeric chains running along the *b*-axis direction (Fig. 2). The distances between the symmetry related Cd^II^ ions [Cd1 ···Cd1^i^; symmetry code (i) = *x*, *y* + 1, *z*] is 7.495 (3) Å.

The O1—Cd1—O2 and O4—Cd1—O5 angles are 53.89 (17)° and 53.88 (18) °, respectively. The corresponding O—M—O (*M* = metal) angles are 52.91 (4)° and 53.96 (4)° in [Cd(C_8_H_5_O_3_)_2_(C_6_H_6_N_2_O)_2_(H_2_O)].H_2_O (Hökelek *et al.*, 2009) and 53.50 (14)° in [Cu_2_(C_8_H_5_O_3_)_4_(C_6_H_6_N_2_O)_4_] (Sertçelik *et al.*, 2013).

The near equality of the C1—O1 [1.262 (9) Å], C1—O2 [1.234 (9) Å] and C9—O4 [1.242 (9) Å], C9—O5 [1.247 (9) Å] bonds in the carboxylate groups indicate delocalized bonding arrangements, rather than localized single and double bonds. The average Cd—O and Cd—N distances are 2.373 (5) and 2.307 (6) Å, respectively, close to standard values (Allen *et al.*, 1987). The Cd atom lies 0.0175 (5) Å and 0.0153 (4) Å below of the carboxylate groups [(O1/O2/C1) and (O4/O5/C9)], respectively. The dihedral angles between the planar carboxylate groups [(O1/O2/C1) and (O4/O5/C9)] and the adjacent benzene rings [A (C2—C7), B (C10/C11A,C12A,C13/C14A/C15A) and B' (C10/C11B/C12B/C13/C14B/C15B)] are 22.7 (8) and 15.6 (10) and 11.4 (11) °, respectively, while the benzene rings, A to B and A to B', are oriented at dihedral angles of 24.2 (7) and 43.0 (8) °, respectively. On the other hand, the pyrazine ring C (N1/N2/C17—C20) is oriented with respect to benzene rings A, B and B' at dihedral angles of 67.5 (4), 89.6 (7) and 86.2 (7) °, respectively.

In the crystal, O–H_water_ ··· O_carboxylate_ hydrogen bonds (Table 1) link adjacent chains into layers parallel to the *bc* plane. The layers are linked *via* C–H_pyrazine_ ··· O_formyl_ hydrogen bonds (Table 1), forming a three-dimensional network.

There is a slipped parallel π-π contact between inversion related benzene rings, A···A^i^, with a centroid-centroid distance of 3.951 (5) Å [normal distance 3.581 (4) Å, slippage 1.668 Å; symmetry code: (i) - *x +1*, - *y*, - *z +1*], and π-π interactions between the disordered benzene rings, B···B^ii^ and B'···B'^ii^ with centroid-centroid distances of 3.870 (11) and 3.873 (12) Å, respectively [symmetry code: (ii) -x, y+1/2, -z+1/2] . There is also a weak C—H···π interaction present (Table 1).

### 2. Experimental

The title compound was prepared by the reaction of CdSO_4_.8/3H_2_O (1.28 g, 5 mmol) in H_2_O (50 ml) and pyrazine (0.80 g, 10 mmol) in H_2_O (30 ml) with sodium 4-formylbenzoate (1.72 g, 10 mmol) in H_2_O (50 ml). The mixture was filtered and set aside to crystallize at ambient temperature for several days, giving plate-like colourless single crystals.

### 3. Refinement

Atoms H71 and H72 (for H_2_O) were located in a difference and refined with a distance restraint: 0-H = 0.82 (2) Å and H···H = 1.35 (2) Å with U_iso_(H) = 1.5U_eq_(O). The C-bound H-atoms were positioned geometrically and constrained to ride on their parent atom: C—H = 0.93 Å with *U*_iso_(H) = 1.2*U*_eq_(C). In one of the two FB anions, the O atom, O6, the aldehyde H atom, H16, and the benzene ring B (C10—C15) are disordered over two positions. The O atoms (O6A and O6B) were freely refined [ratio 0.79 (2):0.21 (2)]. The aldehyde H atoms (H16A and H16B) were refined with a fixed occupancy ratio of 0.8:0.2. The benzene ring atoms [(C11A, H11A, C12A, H12A, C14A, H14A, C15A, H15A) and (C11B, H11B, C12B, H12B, C14B, H14B, C15B, H15B)] were refined with a fixed occupancy ratio of 0.5:0.5.

### Crystal data

**Table:**

[Cd(C_8_H_5_O_3_)_2_(C_4_H_4_N_2_)(H_2_O)]	*F*(000) = 1016
*M**_r_* = 508.76	*D*_x_ = 1.684 Mg m^−^^3^
Monoclinic, *P*2_1_/*c*	Mo *K*α radiation, λ = 0.71073 Å
Hall symbol: -P 2ybc	Cell parameters from 9816 reflections
*a* = 22.6016 (5) Å	θ = 2.7–28.4°
*b* = 7.4947 (2) Å	µ = 1.14 mm^−^^1^
*c* = 11.9196 (3) Å	*T* = 294 K
β = 99.673 (4)°	Plate, colourless
*V* = 1990.38 (9) Å^3^	0.45 × 0.35 × 0.15 mm
*Z* = 4	

### Data collection

**Table:**

Bruker SMART BREEZE CCD diffractometer	3587 independent reflections
Radiation source: fine-focus sealed tube	3497 reflections with *I* > 2σ(*I*)
Graphite monochromator	*R*_int_ = 0.048
φ and ω scans	θ_max_ = 25.3°, θ_min_ = 1.8°
Absorption correction: multi-scan (*SADABS*; Bruker, 2012)	*h* = −27→27
*T*_min_ = 0.625, *T*_max_ = 0.842	*k* = −8→8
40178 measured reflections	*l* = −14→14

### Refinement

**Table:**

Refinement on *F*^2^	Primary atom site location: structure-invariant direct methods
Least-squares matrix: full	Secondary atom site location: difference Fourier map
*R*[*F*^2^ > 2σ(*F*^2^)] = 0.059	Hydrogen site location: inferred from neighbouring sites
*wR*(*F*^2^) = 0.144	H atoms treated by a mixture of independent and constrained refinement
*S* = 1.35	*w* = 1/[σ^2^(*F*_o_^2^) + (0.0316*P*)^2^ + 14.8406*P*] where *P* = (*F*_o_^2^ + 2*F*_c_^2^)/3
3587 reflections	(Δ/σ)_max_ < 0.001
287 parameters	Δρ_max_ = 1.77 e Å^−^^3^
3 restraints	Δρ_min_ = −1.85 e Å^−^^3^

### Special details

**Table:**

Geometry. All esds (except the esd in the dihedral angle between two l.s. planes) are estimated using the full covariance matrix. The cell esds are taken into account individually in the estimation of esds in distances, angles and torsion angles; correlations between esds in cell parameters are only used when they are defined by crystal symmetry. An approximate (isotropic) treatment of cell esds is used for estimating esds involving l.s. planes.
Refinement. Refinement of F^2^ against ALL reflections. The weighted R-factor wR and goodness of fit S are based on F^2^, conventional R-factors R are based on F, with F set to zero for negative F^2^. The threshold expression of F^2^ > 2sigma(F^2^) is used only for calculating R-factors(gt) etc. and is not relevant to the choice of reflections for refinement. R-factors based on F^2^ are statistically about twice as large as those based on F, and R- factors based on ALL data will be even larger.

### Fractional atomic coordinates and isotropic or equivalent isotropic displacement parameters (Å^2^)

**Table:**

	*x*	*y*	*z*	*U*_iso_*/*U*_eq_	Occ. (<1)
Cd1	0.25229 (2)	0.17641 (6)	0.12918 (4)	0.02711 (18)	
O1	0.3237 (2)	0.1914 (8)	0.2983 (4)	0.0451 (13)	
O2	0.3601 (2)	0.1504 (9)	0.1430 (5)	0.0567 (16)	
O3	0.6257 (4)	0.2685 (15)	0.5777 (8)	0.105 (3)	
O4	0.1447 (2)	0.1687 (9)	0.0798 (5)	0.0551 (16)	
O5	0.1829 (2)	0.1597 (8)	0.2594 (5)	0.0503 (15)	
O6A	−0.1449 (4)	0.134 (2)	0.2280 (11)	0.124 (6)	0.79 (2)
O6B	−0.111 (2)	0.139 (9)	0.378 (6)	0.16 (3)	0.21 (2)
O7	0.2521 (2)	0.1713 (7)	−0.0625 (4)	0.0362 (11)	
H71	0.263 (3)	0.278 (4)	−0.054 (7)	0.056*	
H72	0.221 (2)	0.168 (9)	−0.109 (6)	0.056*	
N1	0.2519 (2)	0.4797 (9)	0.1242 (4)	0.0299 (13)	
N2	0.2503 (2)	0.8645 (6)	0.1216 (5)	0.0263 (11)	
C1	0.3678 (3)	0.1700 (9)	0.2472 (6)	0.0330 (15)	
C2	0.4297 (3)	0.1712 (9)	0.3143 (6)	0.0304 (14)	
C3	0.4421 (3)	0.2522 (11)	0.4201 (6)	0.0406 (17)	
H3	0.4112	0.3025	0.4521	0.049*	
C4	0.5005 (4)	0.2584 (12)	0.4780 (6)	0.047 (2)	
H4	0.5089	0.3158	0.5480	0.056*	
C5	0.5460 (3)	0.1806 (12)	0.4330 (7)	0.047 (2)	
C6	0.5336 (4)	0.0950 (14)	0.3294 (8)	0.059 (2)	
H6	0.5645	0.0402	0.2996	0.071*	
C7	0.4760 (3)	0.0897 (12)	0.2694 (7)	0.0452 (19)	
H7	0.4681	0.0321	0.1993	0.054*	
C8	0.6082 (4)	0.1889 (19)	0.4960 (10)	0.082 (4)	
H8	0.6366	0.1221	0.4662	0.099*	
C9	0.1387 (3)	0.1613 (9)	0.1813 (6)	0.0335 (15)	
C10	0.0767 (3)	0.1475 (10)	0.2111 (6)	0.0351 (16)	
C13	−0.0386 (4)	0.1296 (14)	0.2620 (8)	0.054 (2)	
C11A	0.0653 (14)	0.171 (3)	0.320 (3)	0.050 (4)	0.50
H11A	0.0972	0.1880	0.3797	0.060*	0.50
C12A	0.0074 (12)	0.170 (3)	0.342 (2)	0.050 (4)	0.50
H12A	0.0005	0.1985	0.4151	0.060*	0.50
C14A	−0.0278 (12)	0.089 (3)	0.153 (2)	0.050 (4)	0.50
H14A	−0.0592	0.0529	0.0966	0.060*	0.50
C15A	0.0301 (13)	0.104 (3)	0.128 (3)	0.050 (4)	0.50
H15A	0.0369	0.0829	0.0548	0.060*	0.50
C11B	0.0702 (14)	0.106 (4)	0.319 (3)	0.057 (5)	0.50
H11B	0.1038	0.0856	0.3747	0.068*	0.50
C12B	0.0126 (12)	0.093 (3)	0.348 (2)	0.057 (5)	0.50
H12B	0.0076	0.0606	0.4208	0.068*	0.50
C14B	−0.0305 (12)	0.171 (3)	0.153 (2)	0.057 (5)	0.50
H14B	−0.0635	0.1966	0.0976	0.068*	0.50
C15B	0.0255 (13)	0.174 (3)	0.126 (3)	0.057 (5)	0.50
H15B	0.0303	0.1945	0.0515	0.068*	0.50
C16	−0.0996 (5)	0.1259 (19)	0.2908 (12)	0.081 (3)	
H16A	−0.1021	0.1161	0.3677	0.097*	0.80
H16B	−0.1313	0.1104	0.2310	0.097*	0.20
C17	0.2264 (3)	0.5861 (11)	0.0310 (7)	0.0441 (18)	
H17	0.2087	0.5283	−0.0352	0.053*	
C18	0.2260 (3)	0.7705 (9)	0.0313 (6)	0.0391 (17)	
H18	0.2080	0.8306	−0.0339	0.047*	
C19	0.2755 (3)	0.7728 (10)	0.2113 (6)	0.0381 (17)	
H19	0.2933	0.8340	0.2762	0.046*	
C20	0.2764 (3)	0.5877 (9)	0.2121 (6)	0.0375 (16)	
H20	0.2954	0.5323	0.2783	0.045*	

### Atomic displacement parameters (Å^2^)

**Table:**

	*U*^11^	*U*^22^	*U*^33^	*U*^12^	*U*^13^	*U*^23^
Cd1	0.0334 (3)	0.0204 (3)	0.0268 (3)	−0.00118 (19)	0.00312 (19)	−0.00010 (18)
O1	0.031 (3)	0.060 (4)	0.045 (3)	0.007 (2)	0.007 (2)	−0.010 (3)
O2	0.042 (3)	0.092 (5)	0.035 (3)	−0.008 (3)	0.002 (2)	−0.001 (3)
O3	0.068 (5)	0.147 (9)	0.084 (6)	−0.017 (5)	−0.030 (4)	0.003 (6)
O4	0.036 (3)	0.087 (5)	0.043 (3)	0.002 (3)	0.008 (2)	0.009 (3)
O5	0.034 (3)	0.071 (4)	0.045 (3)	−0.006 (3)	0.001 (2)	−0.021 (3)
O6A	0.036 (6)	0.233 (17)	0.102 (10)	0.008 (7)	0.013 (5)	0.026 (10)
O6B	0.10 (4)	0.21 (7)	0.20 (7)	0.00 (4)	0.10 (4)	0.04 (5)
O7	0.044 (3)	0.037 (3)	0.027 (2)	0.000 (2)	0.004 (2)	0.002 (2)
N1	0.010 (2)	0.068 (4)	0.011 (2)	0.000 (2)	−0.0008 (17)	−0.005 (3)
N2	0.035 (3)	0.006 (2)	0.038 (3)	0.002 (2)	0.003 (2)	−0.001 (2)
C1	0.035 (4)	0.023 (4)	0.041 (4)	−0.001 (3)	0.004 (3)	0.002 (3)
C2	0.035 (3)	0.024 (3)	0.033 (3)	−0.003 (3)	0.007 (3)	0.004 (3)
C3	0.039 (4)	0.048 (5)	0.037 (4)	0.003 (3)	0.012 (3)	−0.008 (3)
C4	0.047 (4)	0.060 (6)	0.031 (4)	−0.007 (4)	0.000 (3)	−0.006 (4)
C5	0.034 (4)	0.057 (5)	0.050 (5)	−0.001 (4)	0.001 (3)	0.010 (4)
C6	0.038 (4)	0.078 (7)	0.064 (6)	0.008 (4)	0.014 (4)	−0.006 (5)
C7	0.040 (4)	0.054 (5)	0.043 (4)	0.003 (4)	0.012 (3)	−0.008 (4)
C8	0.042 (5)	0.123 (11)	0.075 (7)	−0.004 (6)	−0.011 (5)	0.002 (7)
C9	0.037 (4)	0.021 (3)	0.042 (4)	0.001 (3)	0.006 (3)	−0.004 (3)
C10	0.034 (4)	0.035 (4)	0.034 (4)	−0.002 (3)	0.002 (3)	−0.003 (3)
C13	0.041 (4)	0.068 (6)	0.057 (5)	0.002 (4)	0.013 (4)	−0.006 (5)
C11A	0.039 (6)	0.070 (11)	0.042 (6)	0.009 (8)	0.006 (4)	0.002 (8)
C12A	0.039 (6)	0.070 (11)	0.042 (6)	0.009 (8)	0.006 (4)	0.002 (8)
C14A	0.039 (6)	0.070 (11)	0.042 (6)	0.009 (8)	0.006 (4)	0.002 (8)
C15A	0.039 (6)	0.070 (11)	0.042 (6)	0.009 (8)	0.006 (4)	0.002 (8)
C11B	0.039 (6)	0.089 (14)	0.040 (6)	0.012 (10)	0.002 (4)	0.004 (10)
C12B	0.039 (6)	0.089 (14)	0.040 (6)	0.012 (10)	0.002 (4)	0.004 (10)
C14B	0.039 (6)	0.089 (14)	0.040 (6)	0.012 (10)	0.002 (4)	0.004 (10)
C15B	0.039 (6)	0.089 (14)	0.040 (6)	0.012 (10)	0.002 (4)	0.004 (10)
C16	0.055 (7)	0.117 (10)	0.075 (7)	−0.005 (6)	0.023 (6)	0.002 (7)
C17	0.045 (4)	0.036 (4)	0.047 (4)	−0.004 (3)	−0.003 (3)	−0.013 (3)
C18	0.049 (4)	0.022 (4)	0.041 (4)	−0.005 (3)	−0.007 (3)	0.005 (3)
C19	0.056 (5)	0.026 (4)	0.030 (4)	−0.011 (3)	−0.002 (3)	0.002 (3)
C20	0.051 (4)	0.023 (4)	0.037 (4)	−0.001 (3)	0.004 (3)	0.006 (3)

### Geometric parameters (Å, º)

**Table:**

Cd1—N1	2.274 (6)	C6—H6	0.9300
Cd1—O7	2.284 (5)	C7—H7	0.9300
Cd1—N2^i^	2.340 (5)	C8—H8	0.9300
Cd1—O1	2.364 (5)	C9—C10	1.505 (10)
Cd1—O5	2.388 (5)	C10—C15A	1.36 (3)
Cd1—O4	2.405 (5)	C10—C11B	1.36 (3)
Cd1—O2	2.423 (6)	C10—C11A	1.38 (3)
Cd1—C9	2.744 (7)	C10—C15B	1.42 (3)
Cd1—C1	2.750 (7)	C13—C12A	1.33 (3)
O1—C1	1.262 (9)	C13—C14B	1.38 (3)
O2—C1	1.234 (9)	C13—C14A	1.40 (3)
O3—C8	1.154 (14)	C13—C12B	1.44 (3)
O4—C9	1.242 (9)	C13—C16	1.475 (13)
O5—C9	1.247 (9)	C11A—C12A	1.38 (3)
O6A—C16	1.165 (15)	C11A—H11A	0.9300
O6A—H16B	0.3504	C12A—H12A	0.9300
O6B—C16	1.12 (6)	C14A—C15A	1.39 (3)
O7—H71	0.83 (2)	C14A—H14A	0.9300
O7—H72	0.82 (2)	C15A—H15A	0.9300
N1—C20	1.365 (9)	C11B—C12B	1.40 (3)
N1—C17	1.410 (10)	C11B—H11B	0.9300
N2—C19	1.318 (9)	C12B—H12B	0.9300
N2—C18	1.326 (9)	C14B—C15B	1.36 (3)
N2—Cd1^ii^	2.340 (5)	C14B—H14B	0.9300
C1—C2	1.491 (9)	C15B—H15B	0.9300
C2—C3	1.385 (10)	C16—H16A	0.9300
C2—C7	1.394 (10)	C16—H16B	0.9300
C3—C4	1.383 (11)	C17—C18	1.382 (11)
C3—H3	0.9300	C17—H17	0.9300
C4—C5	1.368 (12)	C18—H18	0.9300
C4—H4	0.9300	C19—C20	1.387 (10)
C5—C6	1.378 (12)	C19—H19	0.9300
C5—C8	1.479 (12)	C20—H20	0.9300
C6—C7	1.376 (11)		
			
N1—Cd1—O7	89.51 (17)	C6—C7—H7	120.2
N1—Cd1—N2^i^	176.30 (17)	C2—C7—H7	120.2
O7—Cd1—N2^i^	87.02 (18)	O3—C8—C5	127.7 (12)
N1—Cd1—O1	88.49 (18)	O3—C8—H8	116.1
O7—Cd1—O1	137.71 (18)	C5—C8—H8	116.1
N2^i^—Cd1—O1	94.94 (19)	O4—C9—O5	121.6 (7)
N1—Cd1—O5	93.98 (19)	O4—C9—C10	119.4 (6)
O7—Cd1—O5	139.32 (18)	O5—C9—C10	119.0 (6)
N2^i^—Cd1—O5	87.8 (2)	O4—C9—Cd1	61.2 (4)
O1—Cd1—O5	82.94 (17)	O5—C9—Cd1	60.4 (4)
N1—Cd1—O4	91.1 (2)	C10—C9—Cd1	178.3 (5)
O7—Cd1—O4	85.57 (18)	C15A—C10—C11B	116 (2)
N2^i^—Cd1—O4	87.4 (2)	C15A—C10—C11A	118.0 (17)
O1—Cd1—O4	136.69 (18)	C11B—C10—C15B	120.2 (17)
O5—Cd1—O4	53.88 (18)	C11A—C10—C15B	113.1 (19)
N1—Cd1—O2	94.7 (2)	C15A—C10—C9	119.1 (14)
O7—Cd1—O2	84.23 (18)	C11B—C10—C9	119.6 (14)
N2^i^—Cd1—O2	86.3 (2)	C11A—C10—C9	122.9 (15)
O1—Cd1—O2	53.89 (17)	C15B—C10—C9	120.3 (14)
O5—Cd1—O2	135.59 (18)	C12A—C13—C14B	114.6 (19)
O4—Cd1—O2	168.3 (2)	C12A—C13—C14A	119.0 (16)
N1—Cd1—C9	92.74 (18)	C14B—C13—C12B	119.6 (15)
O7—Cd1—C9	112.4 (2)	C14A—C13—C12B	112.0 (17)
N2^i^—Cd1—C9	87.41 (19)	C12A—C13—C16	119.1 (14)
O1—Cd1—C9	109.87 (19)	C14B—C13—C16	120.2 (14)
O5—Cd1—C9	26.99 (19)	C14A—C13—C16	122.0 (14)
O4—Cd1—C9	26.9 (2)	C12B—C13—C16	120.2 (14)
O2—Cd1—C9	161.8 (2)	C12A—C11A—C10	121 (2)
N1—Cd1—C1	91.69 (18)	C12A—C11A—H11A	119.5
O7—Cd1—C1	110.7 (2)	C10—C11A—H11A	119.5
N2^i^—Cd1—C1	90.73 (19)	C13—C12A—C11A	121.2 (19)
O1—Cd1—C1	27.25 (19)	C13—C12A—H12A	119.4
O5—Cd1—C1	109.70 (19)	C11A—C12A—H12A	119.4
O4—Cd1—C1	163.5 (2)	C15A—C14A—C13	119.6 (18)
O2—Cd1—C1	26.64 (19)	C15A—C14A—H14A	120.2
C9—Cd1—C1	136.7 (2)	C13—C14A—H14A	120.2
C1—O1—Cd1	93.7 (4)	C10—C15A—C14A	121 (2)
C1—O2—Cd1	91.7 (4)	C10—C15A—H15A	119.6
C9—O4—Cd1	91.9 (4)	C14A—C15A—H15A	119.6
C9—O5—Cd1	92.6 (4)	C10—C11B—C12B	120 (2)
Cd1—O7—H71	85 (6)	C10—C11B—H11B	120.1
Cd1—O7—H72	123 (6)	C12B—C11B—H11B	120.1
H71—O7—H72	108 (3)	C11B—C12B—C13	119.2 (19)
C20—N1—C17	109.2 (6)	C11B—C12B—H12B	120.4
C20—N1—Cd1	125.0 (4)	C13—C12B—H12B	120.4
C17—N1—Cd1	125.8 (4)	C15B—C14B—C13	120.3 (18)
C19—N2—C18	116.5 (5)	C15B—C14B—H14B	119.9
C19—N2—Cd1^ii^	119.1 (4)	C13—C14B—H14B	119.9
C18—N2—Cd1^ii^	124.4 (4)	C14B—C15B—C10	121 (2)
O2—C1—O1	120.8 (7)	C14B—C15B—H15B	119.5
O2—C1—C2	120.1 (6)	C10—C15B—H15B	119.5
O1—C1—C2	119.1 (6)	O6B—C16—O6A	106 (3)
O2—C1—Cd1	61.7 (4)	O6B—C16—C13	126 (3)
O1—C1—Cd1	59.1 (4)	O6A—C16—C13	127.2 (13)
C2—C1—Cd1	177.9 (5)	O6A—C16—H16A	116.4
C3—C2—C7	119.5 (7)	C13—C16—H16A	116.4
C3—C2—C1	121.2 (6)	O6B—C16—H16B	117.0
C7—C2—C1	119.3 (6)	C13—C16—H16B	117.0
C4—C3—C2	119.9 (7)	H16A—C16—H16B	125.4
C4—C3—H3	120.0	C18—C17—N1	124.4 (7)
C2—C3—H3	120.0	C18—C17—H17	117.8
C5—C4—C3	120.5 (7)	N1—C17—H17	117.8
C5—C4—H4	119.8	N2—C18—C17	122.0 (7)
C3—C4—H4	119.8	N2—C18—H18	119.0
C4—C5—C6	119.7 (7)	C17—C18—H18	119.0
C4—C5—C8	119.7 (9)	N2—C19—C20	122.1 (7)
C6—C5—C8	120.5 (9)	N2—C19—H19	119.0
C7—C6—C5	120.8 (8)	C20—C19—H19	119.0
C7—C6—H6	119.6	N1—C20—C19	125.7 (7)
C5—C6—H6	119.6	N1—C20—H20	117.1
C6—C7—C2	119.5 (7)	C19—C20—H20	117.1
			
N1—Cd1—O1—C1	−96.7 (4)	Cd1—O5—C9—O4	0.4 (7)
O7—Cd1—O1—C1	−9.0 (6)	Cd1—O5—C9—C10	178.2 (5)
N2^i^—Cd1—O1—C1	82.0 (4)	N1—Cd1—C9—O4	87.0 (5)
O5—Cd1—O1—C1	169.1 (5)	O7—Cd1—C9—O4	−3.6 (5)
O4—Cd1—O1—C1	173.4 (4)	N2^i^—Cd1—C9—O4	−89.3 (5)
O2—Cd1—O1—C1	0.2 (4)	O1—Cd1—C9—O4	176.3 (4)
C9—Cd1—O1—C1	171.0 (4)	O5—Cd1—C9—O4	−179.6 (7)
N1—Cd1—O2—C1	84.5 (5)	O2—Cd1—C9—O4	−159.0 (7)
O7—Cd1—O2—C1	173.5 (5)	C1—Cd1—C9—O4	−177.7 (4)
N2^i^—Cd1—O2—C1	−99.1 (5)	N1—Cd1—C9—O5	−93.4 (4)
O1—Cd1—O2—C1	−0.2 (4)	O7—Cd1—C9—O5	176.0 (4)
O5—Cd1—O2—C1	−16.1 (6)	N2^i^—Cd1—C9—O5	90.3 (4)
O4—Cd1—O2—C1	−156.6 (9)	O1—Cd1—C9—O5	−4.0 (5)
C9—Cd1—O2—C1	−29.3 (10)	O4—Cd1—C9—O5	179.6 (7)
N1—Cd1—O4—C9	−94.0 (5)	O2—Cd1—C9—O5	20.6 (9)
O7—Cd1—O4—C9	176.6 (5)	C1—Cd1—C9—O5	2.0 (6)
N2^i^—Cd1—O4—C9	89.4 (5)	O4—C9—C10—C15A	14.6 (15)
O1—Cd1—O4—C9	−5.0 (6)	O5—C9—C10—C15A	−163.3 (13)
O5—Cd1—O4—C9	0.2 (4)	O4—C9—C10—C11B	167.6 (15)
O2—Cd1—O4—C9	146.8 (9)	O5—C9—C10—C11B	−10.3 (16)
C1—Cd1—O4—C9	5.7 (10)	O4—C9—C10—C11A	−167.9 (13)
N1—Cd1—O5—C9	88.2 (4)	O5—C9—C10—C11A	14.2 (15)
O7—Cd1—O5—C9	−5.7 (6)	O4—C9—C10—C15B	−11.1 (16)
N2^i^—Cd1—O5—C9	−88.6 (4)	O5—C9—C10—C15B	171.0 (13)
O1—Cd1—O5—C9	176.2 (5)	C15A—C10—C11A—C12A	−7 (2)
O4—Cd1—O5—C9	−0.2 (4)	C11B—C10—C11A—C12A	−97 (8)
O2—Cd1—O5—C9	−171.0 (4)	C15B—C10—C11A—C12A	17 (3)
C1—Cd1—O5—C9	−178.6 (4)	C9—C10—C11A—C12A	175.2 (14)
O7—Cd1—N1—C20	−150.7 (5)	C14B—C13—C12A—C11A	−29 (2)
O1—Cd1—N1—C20	−13.0 (5)	C14A—C13—C12A—C11A	−1 (3)
O5—Cd1—N1—C20	69.8 (5)	C12B—C13—C12A—C11A	79 (5)
O4—Cd1—N1—C20	123.7 (5)	C16—C13—C12A—C11A	178.4 (16)
O2—Cd1—N1—C20	−66.6 (5)	C10—C11A—C12A—C13	7 (3)
C9—Cd1—N1—C20	96.9 (5)	C12A—C13—C14A—C15A	−4 (2)
C1—Cd1—N1—C20	−40.0 (5)	C14B—C13—C14A—C15A	83 (5)
O7—Cd1—N1—C17	29.5 (5)	C12B—C13—C14A—C15A	−30 (2)
O1—Cd1—N1—C17	167.3 (5)	C16—C13—C14A—C15A	176.7 (15)
O5—Cd1—N1—C17	−109.9 (5)	C11B—C10—C15A—C14A	26 (3)
O4—Cd1—N1—C17	−56.0 (5)	C11A—C10—C15A—C14A	2 (2)
O2—Cd1—N1—C17	113.7 (5)	C15B—C10—C15A—C14A	−81 (6)
C9—Cd1—N1—C17	−82.9 (5)	C9—C10—C15A—C14A	179.9 (14)
C1—Cd1—N1—C17	140.2 (5)	C13—C14A—C15A—C10	3 (3)
Cd1—O2—C1—O1	0.4 (7)	C15A—C10—C11B—C12B	−26 (3)
Cd1—O2—C1—C2	−178.8 (5)	C11A—C10—C11B—C12B	75 (7)
Cd1—O1—C1—O2	−0.4 (8)	C15B—C10—C11B—C12B	−1 (3)
Cd1—O1—C1—C2	178.8 (5)	C9—C10—C11B—C12B	180.0 (16)
N1—Cd1—C1—O2	−97.0 (5)	C10—C11B—C12B—C13	−2 (3)
O7—Cd1—C1—O2	−6.9 (5)	C12A—C13—C12B—C11B	−83 (5)
N2^i^—Cd1—C1—O2	80.2 (5)	C14B—C13—C12B—C11B	2 (3)
O1—Cd1—C1—O2	179.6 (7)	C14A—C13—C12B—C11B	29 (3)
O5—Cd1—C1—O2	168.1 (5)	C16—C13—C12B—C11B	−176.9 (17)
O4—Cd1—C1—O2	163.5 (7)	C12A—C13—C14B—C15B	28 (3)
C9—Cd1—C1—O2	167.2 (4)	C14A—C13—C14B—C15B	−78 (5)
N1—Cd1—C1—O1	83.4 (4)	C12B—C13—C14B—C15B	1 (3)
O7—Cd1—C1—O1	173.5 (4)	C16—C13—C14B—C15B	−180.0 (17)
N2^i^—Cd1—C1—O1	−99.4 (4)	C13—C14B—C15B—C10	−4 (3)
O5—Cd1—C1—O1	−11.5 (5)	C15A—C10—C15B—C14B	90 (7)
O4—Cd1—C1—O1	−16.1 (10)	C11B—C10—C15B—C14B	5 (3)
O2—Cd1—C1—O1	−179.6 (7)	C11A—C10—C15B—C14B	−18 (3)
C9—Cd1—C1—O1	−12.4 (6)	C9—C10—C15B—C14B	−176.8 (16)
O2—C1—C2—C3	157.1 (7)	C12A—C13—C16—O6B	−8 (5)
O1—C1—C2—C3	−22.1 (10)	C14B—C13—C16—O6B	−159 (5)
O2—C1—C2—C7	−22.7 (10)	C14A—C13—C16—O6B	171 (5)
O1—C1—C2—C7	158.2 (7)	C12B—C13—C16—O6B	21 (5)
C7—C2—C3—C4	2.7 (11)	C12A—C13—C16—O6A	160.9 (19)
C1—C2—C3—C4	−177.0 (7)	C14B—C13—C16—O6A	10 (3)
C2—C3—C4—C5	−1.8 (13)	C14A—C13—C16—O6A	−20 (3)
C3—C4—C5—C6	−0.2 (14)	C12B—C13—C16—O6A	−171 (2)
C3—C4—C5—C8	179.8 (9)	C20—N1—C17—C18	−1.0 (10)
C4—C5—C6—C7	1.2 (15)	Cd1—N1—C17—C18	178.8 (6)
C8—C5—C6—C7	−178.8 (10)	C19—N2—C18—C17	0.5 (11)
C5—C6—C7—C2	−0.3 (14)	Cd1^ii^—N2—C18—C17	179.5 (6)
C3—C2—C7—C6	−1.7 (12)	N1—C17—C18—N2	0.2 (13)
C1—C2—C7—C6	178.0 (8)	C18—N2—C19—C20	−0.3 (11)
C4—C5—C8—O3	−7.4 (19)	Cd1^ii^—N2—C19—C20	−179.4 (6)
C6—C5—C8—O3	172.6 (13)	C17—N1—C20—C19	1.2 (10)
Cd1—O4—C9—O5	−0.4 (7)	Cd1—N1—C20—C19	−178.6 (6)
Cd1—O4—C9—C10	−178.2 (6)	N2—C19—C20—N1	−0.6 (13)

Symmetry codes: (i) *x*, *y*−1, *z*; (ii) *x*, *y*+1, *z*.

### Hydrogen-bond geometry (Å, º)

Cg1 is the centroid of the pyrazine ring N1/N2/C17—C20.

**Table:**

*D*—H···*A*	*D*—H	H···*A*	*D*···*A*	*D*—H···*A*
O7—H72···O5^iii^	0.82 (2)	2.10 (6)	2.727 (7)	133 (7)
C18—H18···O6*A*^iv^	0.93	2.52	3.394 (14)	157
C19—H19···O3^v^	0.93	2.43	3.085 (10)	127
C8—H8···*Cg*1^vi^	0.93	2.93	3.691 (10)	147

Symmetry codes: (iii) *x*, −*y*+1/2, *z*−1/2; (iv) −*x*, −*y*+1, −*z*; (v) −*x*+1, −*y*+1, −*z*+1; (vi) −*x*+1, *y*−1/2, −*z*+1/2.
